# Inhibition of Hepatitis C Virus Replication and Viral Helicase by Ethyl Acetate Extract of the Marine Feather Star *Alloeocomatella polycladia*

**DOI:** 10.3390/md10040744

**Published:** 2012-03-28

**Authors:** Atsuya Yamashita, Kazi Abdus Salam, Atsushi Furuta, Yasuyoshi Matsuda, Osamu Fujita, Hidenori Tani, Yoshihisa Fujita, Yuusuke Fujimoto, Masanori Ikeda, Nobuyuki Kato, Naoya Sakamoto, Shinya Maekawa, Nobuyuki Enomoto, Masamichi Nakakoshi, Masayoshi Tsubuki, Yuji Sekiguchi, Satoshi Tsuneda, Nobuyoshi Akimitsu, Naohiro Noda, Junichi Tanaka, Kohji Moriishi

**Affiliations:** 1 Department of Microbiology, Division of Medicine, Graduate School of Medicine and Engineering, University of Yamanashi, 1110 Shimokato, Chuo-shi, Yamanashi 409-3898, Japan; Email: atsuyay@yamanashi.ac.jp (A.Y.); yfujimoto@yamanashi.ac.jp (Y.F.); 2 Radioisotope Center, The University of Tokyo, 2-11-16 Yayoi, Bunkyo-ku, Tokyo 113-0032, Japan; Email: salam_bio26@yahoo.com (K.A.S); tani@ric.u-tokyo.ac.jp (H.T.); akimitsu@ric.u-tokyo.ac.jp (N.A.); 3 Biomedical Research Institute, National Institute of Advanced Industrial Science and Technology (AIST), 1-1-1 Higashi, Tsukuba, Ibaraki 305-8566, Japan; Email: atsushi.furuta@aist.go.jp (A.F.); yellow-3359@hotmail.co.jp (Y.M.); 036.fujita@gmail.com (O.F.); y.sekiguchi@aist.go.jp (Y.S.); noda-naohiro@aist.go.jp (N.N.); 4 Department of Life Science and Medical Bio-Science, Waseda University, 2-2 Wakamatsu-cho, Shinjuku-ku, Tokyo 162-8480, Japan; Email: stsuneda@waseda.jp; 5 University Education Center, University of the Ryukyus, Okinawa, 1 Senbaru, Nishihara, Okinawa 903-0213, Japan; Email: galatheids@yahoo.co.jp; 6 Marine Learning Center, 2-95-101 Miyagi, Chatan, Okinawa 901-0113, Japan; 7 Department of Tumor Virology, Okayama University Graduate School of Medicine, Dentistry, and Pharmaceutical Sciences, Okayama, 2-5-1 Shikata-cho, Okayama 700-8558, Japan; Email: maikeda@md.okayama-u.ac.jp (M.I.); nkato@md.okayama-u.ac.jp (N.K.); 8 Department of Gastroenterology and Hepatology, Tokyo Medical and Dental University, 1-5-45 Yushima, Bunkyo-ku, Tokyo, Japan; Email: nsakamoto.gast@tmd.ac.jp; 9 First Department of Internal Medicine, Faculty of Medicine, University of Yamanashi, Yamanashi, 1110 Shimokato, Chuo-shi, Yamanashi 409-3898, Japan; Email: maekawa@yamanashi.ac.jp (S.M); enomoto@yamanashi.ac.jp (N.E); 10 Institute of Medical Chemistry, Hoshi University, Ebara 2-4-41, Shinagawa-ku, Tokyo 142-8501, Japan; Email: mnakako@hoshi.ac.jp (M.N.); tsubuki@hoshi.ac.jp (M.T.); 11 Department of Chemistry, Biology and Marine Science, University of the Ryukyus, Nishihara, Okinawa 903-0213, Japan

**Keywords:** marine organism, *Alloeocomatella polycladia*, hepatitis C virus, NS3 helicase

## Abstract

Hepatitis C virus (HCV) is a causative agent of acute and chronic hepatitis, leading to the development of hepatic cirrhosis and hepatocellular carcinoma. We prepared extracts from 61 marine organisms and screened them by an *in vitro* fluorescence assay targeting the viral helicase (NS3), which plays an important role in HCV replication, to identify effective candidates for anti-HCV agents. An ethyl acetate-soluble fraction of the feather star *Alloeocomatella polycladia *exhibited the strongest inhibition of NS3 helicase activity, with an IC_50_ of 11.7 µg/mL. The extract of *A. polycladia* inhibited interaction between NS3 and RNA but not ATPase of NS3. Furthermore, the replication of the replicons derived from three HCV strains of genotype 1b in cultured cells was suppressed by the extract with an EC_50_ value of 23 to 44 µg/mL, which is similar to the IC_50_ value of the NS3 helicase assay. The extract did not induce interferon or inhibit cell growth. These results suggest that the unknown compound(s) included in *A. polycladia* can inhibit HCV replication by suppressing the helicase activity of HCV NS3. This study may present a new approach toward the development of a novel therapy for chronic hepatitis C.

## 1. Introduction

Hepatitis C virus (HCV) is an etiological agent of liver disease including steatosis, cirrhosis, and hepatocellular carcinoma, and has infected over 170 million individuals worldwide [1,2]. HCV belongs to the genus *Hepacivirus* of the *Flaviviridae* family. The genome of HCV is a single positive-strand RNA composed of 9.6 kb flanked by 5' and 3'-untranscribed regions (UTRs) and encodes a polyprotein consisting of approximately 3000 amino acids [3]. The polyprotein is translated from a viral genome by an internal ribosome entry site (IRES), which is localized in 5'-UTR [4]. The translated polyprotein is cleaved by host and viral proteases into 10 proteins. The structural proteins consisting of core, E1, and E2 and a viroporin p7, which has not yet been classified as either a structural or nonstructural protein, are located in the *N*-terminal quarter of the polyprotein. The nonstructural proteins including NS2, NS3, NS4A, NS4B, NS5A, and NS5B occupy the remaining portion of the polyprotein and form a replication complex with several host factors.

HCV NS3 is well known to play a crucial role in viral replication because it possesses helicase and protease activities [5,6]. The *N*-terminal third of NS3 forms a complex with the NS4A protein and exhibits serine protease activity (NS3-4A protease) to cleave the viral polyprotein for the maturation of viral proteins [7]. The remaining portion of NS3 occupies the RNA helicase domain, characterized by the activities of ATPase and RNA binding, both of which contribute to the unwinding of duplex RNA [8,9]. The helicase activity is needed to separate duplex RNA during viral RNA replication [10]. A negative-strand RNA is synthesized based on a viral genome (positive strand) after the uncoating of a viral particle in the infected cells and then is itself used as a template to synthesize a positive-strand RNA packaged into the viral particle. Thus, helicase as well as protease activities of NS3 can be targeted for use in the development of antiviral agents against HCV.

The current therapy, which combines pegylated interferon with ribavirin, is effective in only about half of patients infected with the most common genotype worldwide, genotype 1 [11,12,13]. However, this therapy has side effects including influenza-like symptoms, cytopenias, and depression [11]. Furthermore, no effective vaccines for HCV have been developed yet. Biotechnological advances of the past decade have led to the development of novel therapies using anti-HCV agents that directly target HCV proteins or host factors required for HCV replication. This approach has been named either “specifically targeted antiviral therapy for hepatitis C” (STAT-C) or “directed-acting antiviral agents” (DAA) [14,15,16]. Several compounds of STAT-C or DAA have proceeded to clinical trials. Telaprevir and boceprevir, which are categorized as advanced NS3/4A protease inhibitors, were recently approved for the treatment of chronic hepatitis C patients infected with genotype 1 in the US, EU, Canada, and Japan [17,18]. However, the emergence of drug-resistant viruses is the major problem for therapies using antiviral compounds [19,20]. Accordingly, several kinds of drugs targeting various molecules or positions will be required for the complete eradication of the virus from hepatitis C patients.

The helicase activity of NS3 could be targeted by development of anti-HCV compound in addition to its protease activity. Belon *et al*. reported that 1-*N*,4-*N*-*bis*[4-(1*H*-benzimidazol-2-yl)phenyl]benzene-1,4-dicarboxamine, designated as (BIP)_2_B, is a potent and selective inhibitor of HCV NS3 helicase [21]. (BIP)_2_B could not affect ATP hydrolysis without RNA or at a saturated concentration of RNA. QU663 inhibits the unwinding activity of NS3 helicase by binding to the RNA-binding groove irrespective of its own ATPase activity [22]. Compound QU663 may competitively bind the RNA-binding site of NS3 but not affect ATPase activity, resulting in the inhibition of unwinding activity.

Various drugs have been generated from natural products, especially those from terrestrial plants and microbes. The development of drugs from natural products has declined in the past two decades by the emergence of high-throughput screening of synthetic chemical libraries. However, recent technical advances in the determination of molecular structures and in the synthesis of chemical compounds have raised awareness about natural products as a resource for drug development [23,24,25]. Several groups recently reported natural products that inhibit HCV replication *in vitro*. For instance, silbinin, which is identified from the milk thistle [26,27], epigallocatechin 3-gallate, which is from green tea [28], and proanthocyanidins, which are from blueberry leaves [29], can inhibit HCV replication in cultured cells. Marine organisms including plants and animals were recently established as a representative natural resource library for drug development, since there are estimated to be more than 300,000 species of marine organisms. The products isolated from the marine organisms often possess potent biological activities corresponding to the organisms’ own novel molecular structures. Thus, marine natural products are considered to include highly significant lead compounds for drug development [30,31]. For example, trabectedin (Yondelis), cytarabine (Ara-C), and eribulin (Halaven) are approved anticancer drugs developed from marine organisms [32]. However, marine organisms have not yet been screened for development into anti-HCV agents.

In this study, we screened extracts of marine organisms by using an *in vitro* fluorescence NS3 helicase assay and HCV replicon system to find candidates for safe and effective anti-HCV agents. The marine feather star *Alloeocomatella polycladia* may produce anti-HCV helicase agents that suppress HCV replication.

## 2. Results and Discussion

### 2.1. Primary Screening of Marine Organism Extracts on HCV NS3 Helicase Activity

**Figure 1:**
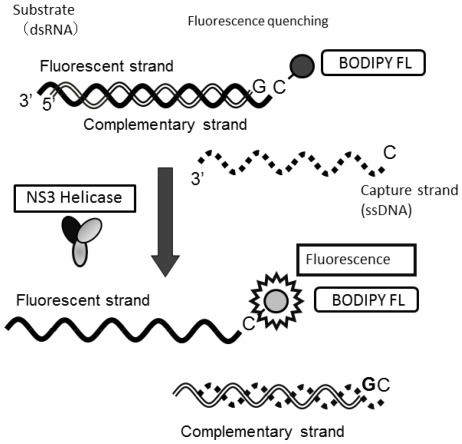
Schematic representation of the PET assay system for unwinding activity of HCV NS3 helicase. The fluorescent dye (BODIPY FL) is attached to the cytosine at the 5'-end of the fluorescent strand and quenched by the guanine base at the 3'-end of the complementary strand via photoinduced electron transfer. When the helicase unwinds the double-strand RNA substrate, the fluorescence of the dye emits bright light upon the release of the dye from the guanine base. The capture strand, which is complementary to the complementary strand, prevents the reannealing of the unwound duplex.

**Figure 2:**
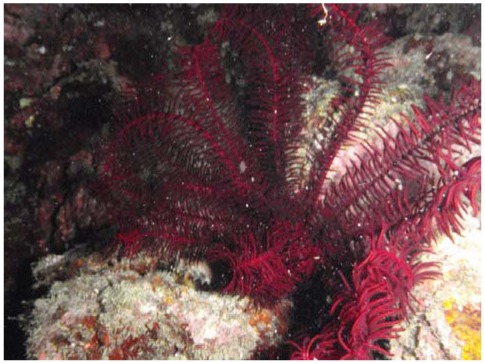
*Alloeocomatella polycladia *belongs to a class of feather star (Echinodermata, Crinoidea). The ethyl acetate fraction prepared from the marine organism was designated SG1-23-1 in this study.

**Figure 3:**
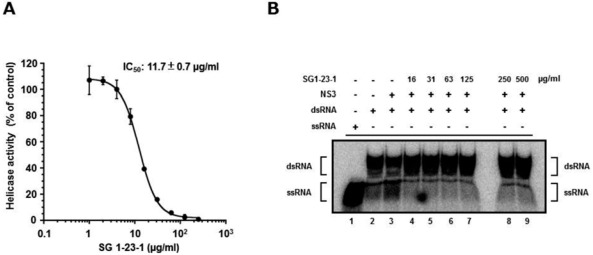
Effect of SG1-23-1 on the unwinding activity of NS3 helicase. (**A**) NS3 helicase activity was measured by PET assay. The reactions were carried out in the absence or presence of SG1-23-1. Helicase activity in the absence of SG1-23-1 was defined as 100% helicase activity. Each value represents the mean of three independent reactions. Error bars indicate standard deviation. The data represent three independent experiments. (**B**) The unwinding activity of NS3 helicase was measured by RNA unwinding assay using radioisotope-labeled RNA. The heat-denatured single-strand RNA (26-mer) and the partial duplex RNA substrate were applied to lanes 1 and 2, respectively. The duplex RNA was reacted with NS3 (300 nM) in the presence of SG1-23-1 (lanes 4 to 9, 16 to 500 µg/mL). The resulting samples were subjected to native polyacrylamide gel electrophoresis.

We employed high-throughput screening using a photoinduced electron transfer (PET) assay to identify inhibitors of HCV NS3 helicase activity from extracts of marine organisms (Figure 1). The EtOAc- and MeOH-soluble extracts were prepared from marine organisms obtained from the sea around Okinawa Prefecture, Japan. We identified 16 extracts possessing an arbitrary level of inhibitory activity, which is defined as below 60% of the control in this study (Table 1). Five extracts exhibited high inhibition levels (<30%), and eleven extracts exhibited intermediate inhibition levels (30% to 60%). The EtOAc extract prepared from the feather star *Alloeocomatella polycladia *(Figure 2) exhibited the strongest inhibitory activity among them, and was designated SG1-23-1 in this study. Treatment with SG1-23-1 inhibited the helicase activity in a dose-dependent manner (Figure 3A). The value of IC_50_ is calculated as 11.7 ± 0.7 µg/mL. We confirmed the effect of SG1-23-1 on NS3 helicase unwinding activity by the RNA helicase assay using ^32^P-labeled double-stranded RNA (dsRNA) as a substrate. Treatment with SG1-23-1 inhibited dsRNA dissociation at concentrations of 16 µg/mL and above (Figure 3B). These results suggest that treatment with SG1-23-1 inhibits the unwinding ability of HCV NS3 helicase.

**Table 1:** Inhibitory effects of marine organism extracts on hepatitis C virus (HCV) NS3 helicase activity.

	Helicase Activity				
Sample	(% of control)	Specimen	Phylum	Extract	Collection Site
OK-99-2	78	*Agelas* sp.	Porifera	EtOAc	Shimoji Island
OK-99-3	73	*Plakortis* sp.	Porifera	EtOAc	Shimoji Island
OK-99-4	60	*Dysidea arenaria*	Porifera	EtOAc	Shimoji Island
OK-99-5	96	*Theonella cupola*	Porifera	EtOAc	Shimoji Island
OK-99-6	52	*Theonella conica*	Porifera	EtOAc	Shimoji Island
OK-99-7	85	*Epipolasis kushimotoensis*	Porifera	EtOAc	Shimoji Island
OK-99-9	51	*Hyrtios* sp.	Porifera	EtOAc	Shimoji Island
OK-99-10	75	*Theonella* sp.	Porifera	EtOAc	Shimoji Island	
OK-99-12	53	*Isis hippuris*	Cnidaria	EtOAc	Shimoji Island	
OK-99-13	68	*Acanthella* sp.	Porifera	EtOAc	Shimoji Island	
OK-99-15	64	*Phyllospongia* sp.	Porifera	EtOAc	Shimoji Island	
OK-99-17	59	*Petrosia* sp.	Porifera	EtOAc	Shimoji Island	
OK-99-18	80	*Fasciospongia rimosa*	Porifera	EtOAc	Shimoji Island	
OK-99-20	77	*Echinoclathria* sp.	Porifera	EtOAc	Shimoji Island	
OK-99-21	68	*Strongylophora *sp.	Porifera	EtOAc	Shimoji Island	
OK-99-23	74	*Dysidea herbacea*	Porifera	EtOAc	Shimoji Island	
OK-99-26	55	*Dysidea* cf. *arenaria*	Porifera	EtOAc	Shimoji Island	
OK-99-28	123	*Plakortis* sp.	Porifera	EtOAc	Shimoji Island	
OK-99-31	118	*Spongia* sp.	Porifera	EtOAc	Okinawa Island	
OK-99-34	119	*Theonella swinhoei*	Porifera	EtOAc	Okinawa Island	
OK-99-35	108	*Petrosia *sp.	Porifera	EtOAc	Okinawa Island	
OK-99-36	90	*Acanthella* sp.	Porifera	EtOAc	Okinawa Island	
OK-99-37	102	*Luffariella* sp.	Porifera	EtOAc	Okinawa Island	
OK-99-41	62	*Dysidea* cf. *arenaria*	Porifera	EtOAc	Okinawa Island	
OK-99-43	85	*Xestospongia* sp.	Porifera	EtOAc	Okinawa Island	
OK-99-44	61	*Dysidea arenaria*	Porifera	EtOAc	Okinawa Island	
OK-99-47	108	*Dysidea* cf. *arenaria*	Porifera	EtOAc	Okinawa Island	
OK-99-49	90	*Petrosia* sp.	Porifera	EtOAc	Chibishi	
OK-99-51	69	*Isis hippuris*	Cnidaria	EtOAc	Chibishi	
OK-99-52	78	*Petrosia *sp.	Porifera	EtOAc	Kuro Island	
OK-99-55	65	*Acanthella* sp.	Porifera	EtOAc	Kuro Island	
OK-99-57	84	*Theonella swinhoei*	Porifera	EtOAc	Kuro Island	
OK-99-63	117	*Epipolasis kushimotoensis*	Porifera	EtOAc	Kuro Island	
OK-99-64	98	*Xestospongia* sp.	Porifera	EtOAc	Kuro Island	
SG1-1-2	77	*Comanthus gisleni*	Echinodermata	MeOH	Kume Island	
SG1-2-2	112	*Stephanometra indica*	Echinodermata	MeOH	Kume Island	
**SG1-5-2**	33	*Comantella* sp. cf. *maculata*	Echinodermata	MeOH	Kume Island	
SG1-9-2	57	*Phanogenia gracilis*	Echinodermata	MeOH	Kume Island	
**SG1-12-2**	39	*Comanthus parvicirrus*	Echinodermata	MeOH	Kume Island	
SG1-14-2	117	*Comaster schlegelii*	Echinodermata	MeOH	Kume Island	
**SG1-15-2**	26	Colobometridae sp.	Echinodermata	MeOH	Kume Island	
SG1-16-2	66	*Cenometra bella*	Echinodermata	MeOH	Kume Island	
SG1-19-2	78	*Comaster nobilis*	Echinodermata	MeOH	Kume Island	
**SG1-21-2**	32	*Oxycomanthus *sp.	Echinodermata	MeOH	Kume Island	
**SG1-23-1**	-3	*Alloeocomatella polycladia *	Echinodermata	EtOAc	Kume Island	
**SG1-24-1**	24	*Comanthus *sp.	Echinodermata	EtOAc	Kume Island	
**SG1-26-2**	51	*Oxycomanthus benetti*	Echinodermata	MeOH	Kume Island	
**SG1-28-2**	38	*Lamprometra palmata*	Echinodermata	MeOH	Kume Island	
**SG1-30-1**	25	*Colobometra perspinosa*	Echinodermata	EtOAc	Kume Island	
**SG1-31-1**	26	*Comanthus *sp.	Echinodermata	EtOAc	Kume Island	
**SG1-33-1**	32	*Basilometra boschmai*	Echinodermata	EtOAc	Kume Island	
SG3-1	82	*Stereonephthya *sp.	Cnidaria	EtOAc	Tokashiki Island	
SG3-4	73	*Dysidea* cf. *arenaria*	Porifera	EtOAc	Tokashiki Island	
SG3-6	74	*Stylotella *sp.	Porifera	EtOAc	Tokashiki Island	
SG3-10	139	*Epipolasis* sp.	Porifera	EtOAc	Tokashiki Island	
SG3-11	97	*Nephthea* sp.	Cnidaria	EtOAc	Tokashiki Island	
SG3-21	106	*Myrmekioderma* sp.	Porifera	EtOAc	Tokashiki Island	
SG3-25	111	*Pseudoceratina purpurea*	Porifera	EtOAc	Tokashiki Island	
SG3-26	95	*Leucetta *sp.	Porifera	EtOAc	Tokashiki Island	
SG3-28	65	*Lyngbya *sp.	Cyanobacteria	EtOAc	Tokashiki Island	
SG3-29	61	*Dysidea *sp.	Porifera	EtOAc	Tokashiki Island	

Total number of marine organisms: 61; Marine organisms that strongly inhibit NS3 helicase activity (<30%) (boldface and underlined): 5; Extracts of organisms that exhibit intermediate inhibition of NS3 helicase activity (30%–60%) (underlined): 11; EtOAc: Ethyl acetate; MeOH: Methanol.

### 2.2. Effect of SG1-23-1 on HCV NS3 ATPase and RNA Binding Activities

**Figure 4:**
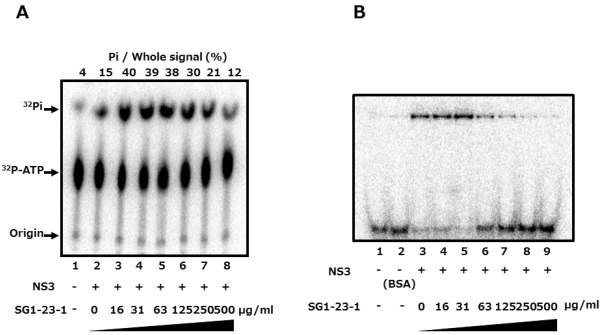
Effect of SG1-23-1 on ATPase and RNA-binding activities of NS3 helicase.(**A**) The reaction mixtures were incubated with [γ-^32^P] ATP as described in Materials and Methods. The reaction mixtures were subjected to thin-layer chromatography. The start positions and migrated positions of ATP and free phosphoric acid are indicated as “Origin”, “^32^P-ATP”, and “^32^P-Pi”, respectively, on the left side of this figure. The data represent three independent experiments. (**B**) Gel mobility shift assay for RNA-binding activity of NS3 helicase. The reaction was carried out at the indicated concentration of SG1-23-1. The reaction mixture was subjected to gel mobility shift assay. The data represent three independent experiments.

The unwinding ability of HCV helicase is dependent on ATP binding, ATP hydrolysis, and RNA binding [8,9]. We examined the effect of SG1-23-1 on the ATPase activity of NS3 helicase. The ratio of free phosphate (^32^P-Pi) in ATP (^32^P-ATP) was measured in the presence of SG1-23-1. The reaction was carried out between 16 and 500 µg of SG1-23-1 per milliliter. ATPase activity was slightly increased at 16 µg SG1-23-1 per milliliter and slightly decreased at 500 µg SG1-23-1 per milliliter (Figure 4A). However, the helicase activity was decreased to less than 10% in the presence of 50 µg of SG1-23-1 per milliliter (Figure 3A,B). Next, we examined the effect of SG1-23-1 on the binding of NS3 helicase to single-strand RNA (ssRNA). A gel-mobility shift assay was employed to estimate the binding activity of NS3 to 21 mer of ssRNA. The binding of NS3 to ssRNA was inhibited with SG1-23-1 in a dose-dependent manner (Figure 4B). These results suggest that SG1-23-1 contains the compound that inhibits RNA binding to NS3 helicase.

### 2.3. Effect of SG1-23-1 on HCV RNA Replication in HCV 1b Replicon Cells

**Figure 5:**
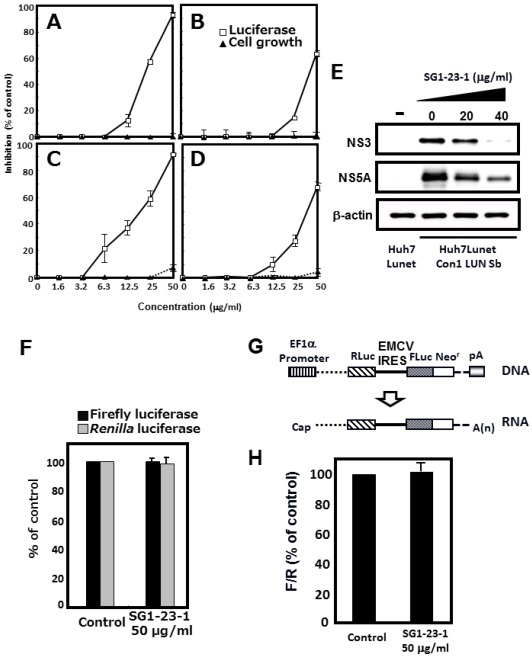
Effect of SG1-23-1 on viral replication in replicon cell lines. (**A**–**D**) Huh7 Lunet/Con1 LUN Sb #26 (**A**), Huh7 rep Feo (**B**), Huh7#94/ORN3-5B#24 (**C**), and OR6 (**D**) cell lines were incubated in medium containing various concentrations of SG1-23-1. Luciferase and cytotoxicity assays were carried out as described in Materials and Methods. Error bars indicate standard deviation. The data represent three independent experiments. (**E**) Protein extract was prepared from Huh7 Lunet/Con1 LUN Sb #26 cells treated for 72 h with an indicated concentration of SG1-23-1 and then was subjected to Western blotting using antibodies to NS3, NS5A, and beta-actin. (**F**) Huh7 cell line transfected with pEF Fluc IN vector or pEF Rluc IN was established in the presence of G418. Both cell lines were incubated without (control) and with 50 μg/mL SG1-23-1. Firefly or *Renilla *luciferase activity was measured 72 h post-treatment. Luciferase activity was normalized with protein concentration. Error bars indicate standard deviation. The data represent three independent experiments. (**G**) Schematic structure of the plasmid, pEFRluc EMCV IRES Feo. The bicistronic gene is transcribed under the control of elongation factor 1α (EF1α) promoter. The upstream cistron encoding *Renilla* luciferase (RLuc) is translated by a cap-dependent mechanism. The downstream cistron encodes the fusion protein (Feo), which consists of the firefly luciferase (Fluc) and neomycin phosphotransferase (Neo^r^), and is translated under the control of the EMCV IRES. (**H**) Huh7 cell line transfected with pEF Rluc EMCV IRES Feo was established in the presence of G418. The cells were incubated for 72 h without (control) and with 50 µg/mL of SG1-23-1. Firefly or *Renilla *luciferase activity was measured by the method described in Materials and Methods and was normalized by the protein concentration. F/R: Relative ratio of Firefly luciferase activity to *Renilla *luciferase activity. F/R is presented as a percentage of the control condition. Error bars indicate standard deviation. The data represent three independent experiments.

We investigated the effect of SG1-23-1 on both viral replication and growth of the replicon cell lines. The cell lines possess viral subgenomic RNAs derived from three genotype 1b strains (strains N [33], Con1 [34], and O [35]) or a full genomic RNA derived from the O strain [35]. Each cell line was treated with various concentrations of SG1-23-1. The treated cells were harvested 72 h post-treatment. Treatment with SG1-23-1 suppressed HCV RNA replications of all cell lines in a dose-dependent manner irrespectively of full- and sub-genome replicons; it exhibited no effect below 25 µg/mL and little effect on cellular viability at the highest concentration, 50 µg/mL (Figure 5C,D). Both HCV NS3 and NS5A were decreased at the protein level in a dose-dependent manner, corresponding to the viral replication, but beta-actin was not changed in the cell line harboring subgenome replicon RNA of the Con1 strain (Figure 5E).

The inhibitory effect of SG1-23-1 on HCV replication is summarized in Table 2. The inhibitory effects on the HCV replication of the subgenome replicon derived from Con1, O, and N strains were 22.9 ± 0.4, 19.9 ± 1.8, and 44.2 ± 1.5 µg/mL, respectively, as EC_50_; and 48.1 ± 1.5, 48.5 ± 0.3, and >50 µg/mL, respectively, as EC_90_. Treatment with SG1-23-1 inhibited the replication of the subgenome replicon of the O strain (EC_50_: 19.9 ± 1.8 µg/mL; EC_90_: 48.5 ± 0.3 µg/mL) at a more potent level than the replication of the full genomic replicon of the O strain (EC_50_: 39.5 ± 0.8 µg/mL; EC_90_: >50 µg/mL). When luciferase of firefly or *Renilla* was expressed under the control of the EF promoter, neither showed a significant change in activity in the presence of SG1-23-1 (Figure 5F). The replicon RNA of HCV is composed of the 5'-UTR of HCV, indicator genes (luciferase and drug-resistant genes), encephalomyocarditis virus (EMCV) IRES, the viral genes encoding complete or nonstructural proteins, and the 3'-UTR of HCV in that order [33,34,35]. The replicon RNA replicated autonomously in several HCV replication-permissive cell lines derived from several hepatoma cell lines. Nonstructural proteins in replicon cells were polycistronically translated through EMCV IRES. The cap-dependent translated mRNA, including *Renilla* luciferase, EMCV IRES, and the firefly luciferase/neomycin-resistant gene in that order, was constructed to examine the effect of the extract on EMCV-IRES-dependent translation (Figure 5G). When the expression of the mRNA was transcribed by an EF promoter of the transfected plasmid in the presence of SG1-23-1, the ratio of firefly luciferase activity to *Renilla* luciferase activity was not changed, suggesting that treatment with SG1-23-1 exhibited no effect on EMCV-IRES-dependent translation (Figure 5H). Thus, the inhibitory effect of SG1-23-1 on the luciferase activity must correspond to the replication efficiency of the replicon RNA but not to the inhibition of luciferase activity or the inhibition of EMCV-IRES-dependent translation. The inhibitory effect of the extract on the viral replication is similar to that of the extract on the helicase activity with respect to the values of IC_50_ and EC_50_ (Figure 3A and Table 2). These results suggest that treatment with SG1-23-1 inhibits HCV replication in a manner similar to that of the inhibitory effect on NS3 helicase activity.

**Table 2:** Anti-HCV activity of SG1-23-1 in different replicon cell lines of genotype 1b.

Replicon Cell Line		Virus Strain	EC_50 _^a^	EC_90 _^b^	CC_50 _^c^
		(Genotype 1b)	(µg/mL)	(µg/mL)	(µg/mL)
Subgenome					
	Huh7 Lunet/Con1 LUN Sb #26	Con1	22.9 ± 0.4	48.1 ± 1.5	>50
	Huh7 rep Feo	N	44.2 ± 1.5	>50	>50
	Hu7#94/ORN3-5B#24	O	19.9 ± 1.8	48.8 ± 0.3	>50
Full genome					
OR6		O	39.5 ± 0.8	>50	>50

All data represent means ± standard deviation for three independent experiments; ^a^ Fifty percent effective concentration based on the inhibition of HCV replication; ^b^ Ninety percent effective concentration based on the inhibition of HCV replication; ^c^ Fifty percent cytotoxicity concentration based on the reduction of cell viability.

### 2.4. Effect of SG1-23-1 on the Interferon (IFN) Signaling Pathway

**Figure 6:**
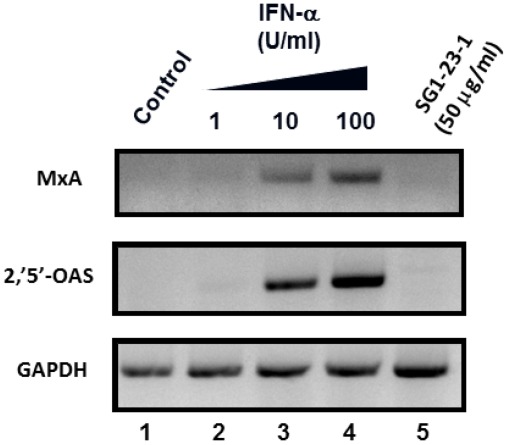
Effect of SG1-23-1 on interferon signaling pathway. Huh7 Lunet/Con1 LUN Sb #26 cells were treated without (lane 1) or with 1, 10, or 100 U/mL IFNa-2b (lanes 2–4), and 50 µg/mL SG1-23-1 (lane 5) for 48 h. The mRNAs of MxA, 2',5'-OAS, and glyceraldehyde-3-phosphate dehydrogenase (GAPDH) as an internal control were detected by reverse-transcription polymerase chain reaction (RT-PCR). Error bars indicate standard deviation. The data represent three independent experiments.

It has been reported that the HCV replication in cultured cells is potently inhibited by interferon (IFN) [36,37]. We examined whether or not treatment with SG1-23-1 induces interferon from replicon cells. The replicon cells were treated with various concentrations of interferon-alpha 2b or 50 µg of SG1-23-1 per milliliter. The treated cells were harvested at 72 h post-treatment. The interferon-inducible genes, MxA and 2',5'-OAS, were induced with IFN-alpha 2b but not with SG1-23-1 (Figure 6). These results suggest that the inhibitory effect of SG1-23-1 on the replication of the HCV replicon is independent of the IFN signaling pathway.

Treatment with SG1-23-1 suppressed the helicase activity of NS3 in a dose-dependent manner and exhibited an IC_50_ of 11.7 µg/mL. Interestingly, treatment with SG1-23-1 inhibited the RNA binding activity of the helicase but not the ATPase activity of NS3. Treatment with SG1-23-1 inhibited the luciferase activity corresponding to the HCV replication in the replicon cell lines, but not the enzymatic activity of luciferase or the translational activity of EMCV IRES, suggesting that treatment with SG1-23-1 decreases HCV replication. Figure 4 shows that the viral proteins NS3 and NS5A in replicon cells were decreased by treatment with SG1-23-1, supporting the notion that SG1-23-1 inhibits HCV replication but not the enzymatic activity of luciferase. The inhibition of cell growth would not contribute to the inhibition of HCV replication by SG1-23-1 (Figure 3 and Table 2). Treatment with SG1-23-1 did not induce the interferon-stimulated genes in the replicon cell lines (Figure 6), suggesting that inhibition of HCV replication by treatment with SG1-23-1 is not due to interferon induction or interferon signaling. The extract SG1-23-1 inhibited the HCV replicon with an EC_50_ of 22 to 44 µg/mL, which is similar to the value of IC_50_. These results suggest that the anti-HCV compound(s) included in *A. polycladia *can suppress viral replication by inhibiting NS3 helicase activity.

## 3. Experimental Section

### 3.1. Preparation of Extracts from Marine Organisms

All marine organisms used in this study were collected by hand during scuba diving off Shimoji, Okinawa, Chibishi, Kuro, Kume, and Tokashiki Islands in Okinawa Prefecture, Japan. In the case of OK-99-tagged extract, a specimen was soaked in ethanol. The ethanol-soluble fraction was concentrated, and the resulting aqueous material was suspended in ethyl acetate (EtOAc). The organic fraction was used for screening.

Each specimen from Kume was soaked in ethanol. The ethanol-soluble fraction was concentrated. The resulting material was suspended in EtOAc. The EtOAc-soluble fraction was used for screening and tagged with SG1 and the last digit of “1”. The water layer was concentrated to dryness and suspended in methanol (MeOH). The MeOH-soluble fraction was used for screening and tagged with SG1 and the last digit of “2”.

Each specimen from Tokashiki was extracted three times with acetone. After removal of acetone from the solution, the residual material was suspended in EtOAc. The EtOAc-soluble fraction was used for screening and tagged with SG3.

All samples were dried and then solubilized in dimethyl sulfoxide (DMSO) before testing.

### 3.2. High-Throughput Screening of NS3 Helicase Inhibitors

A continuous fluorescence assay based on photoinduced electron transfer (PET) was described previously [38] and was slightly modified with regard to the reaction mixture. A schematic diagram of the PET assay for HCV NS3 helicase activity is shown in Figure 1. The double-strand RNA was prepared as a substrate by annealing, at a 1:2 molar ratio, a 5' BODIPY FL-labeled 37-mer (5'-CUAUUACCUCCACCCUCAUAACCUUUUUUUUUUUUUU-3') to a 23-mer (GGUUAUGAGGGUGGAGGUAAUAG). When unwound by HCV NS3 helicase, the unlabeled ssRNA was captured by a DNA capture strand (5'-CTATTACCTCCACCCTCATAACC-3'). A fluorescent-dye-labeled oligonucleotide was purchased from J-Bio 21 Corporation. BODIPY FL was attached to the 5'-end via an aminohexylphosphate linker with a six-carbon spacer. Unlabeled oligonucleotides were purchased from Japan Bio Services Co., Ltd. The PET NS3 helicase assay was carried out in 22 µL of 25 mM MOPS-NaOH (pH 6.5) containing 3 mM MgCl_2_, 2 mM dithiothreitol (DTT), 4 U RNasin, 50 nM of the double-strand RNA described above, 100 nM DNA capture strand, 5 mM ATP, and the extract (25 µg/mL) and 240 nM HCV NS3 helicase. The reaction was started by the addition of HCV NS3 helicase. The reaction mixture was incubated at 37 °C for 30 min. The fluorescence intensity was recorded every 5 s until 5 min post-reaction, and then every 30 s between 5 and 30 min post-reaction by using a LightCycler 1.5 (Roche Diagnostics, Tokyo, Japan). The initial reaction velocity was calculated and represented as NS3 helicase activity.

### 3.3. ATPase Assay

NS3 ATPase activity was determined by the method of Gallinari *et al.* [39] with slight modifications. The reaction was carried out at 37 °C for 10 min in 10 μL of the reaction mixture containing 25 mM MOPS-NaOH (pH 7.0), 1 mM DTT, 5 mM MgCl_2_, 5 mM CaCl_2_, 1 mM [γ-^32^P] ATP (Muromachi, Tokyo, Japan), 300 nM NS3, and 0.1 μg poly (U) per microliter and an indicated concentration of SG1-23-1, and then was terminated by the addition of 15 microliters of 10 mM EDTA. Two microliters of the reaction mixture were spotted onto a polyethyleneimine cellulose sheet (Merck, Darmstadt, Germany) and then developed in 0.75 M LiCl/1 M formic acid solution at room temperature for 20 min. The sheet was air-dried completely and then exposed to an image plate. Radioactive bands were visualized with an Image Reader FLA-9000 and quantified by Multi Gauge V 3.11 software (version 3.11; Fujifilm: Tokyo, Japan, 2008).

### 3.4. RNA Helicase Assay

NS3 RNA helicase assay was carried out by the method of Gallinari *et al.* [39] with slight modifications. The substrate for annealing two complementary RNA oligonucleotides, 5'-AGAGAGAGAGGUUGAGAGAGAGAGAGUUUGAGAGAGAGAG-3' (40-mer, template strand) and 5'-CAAACUCUCUCUCUCUCAACAAAAAA-3' (26-mer, release strand) was purchased from Shanghai GenePharma Co., Ltd. The release strand was labeled at the 5'-end with [γ-^32^P] ATP (Muromachi, Tokyo, Japan) using the T4 polynucleotide kinase (Toyobo, Osaka, Japan) at 37 °C for 60 min and then purified by phenol chloroform extraction. The template and the labeled release strands were annealed at a molar ratio of 3:1 (template: release), denatured at 80 °C for 5 min, and slowly renatured at 23 °C for 30 min in an annealing buffer consisting of 20 mM Tris-HCl (pH 8), 0.5 M NaCl, and 1 mM EDTA. The partial duplex RNA substrate was purified on a G-50 micro column (GE Healthcare, Uppsala, Sweden) and stored at −20 °C in H_2_O containing 0.25 U of RNasin Plus (Promega, Madison, WI, USA) per microliter.

SG1-23-1 extract was added at various concentrations to a helicase reaction mixture consisting of 25 mM MOPS-NaOH (pH 7.0), 2.5 mM DTT, 2.5 U of RNasin Plus (Promega), 100 μg of BSA per milliliter, and 3 mM MgCl_2_. The mixture was supplemented with 300 nM NS3 protein and 5 fM ^32^P-labeled partial duplex RNA substrate. It was then preincubated at 23 °C for 15 min. After adding ATP at a final concentration of 5 mM, the reaction mixture (20 µL) was incubated at 37 °C for 30 min and stopped by adding 5 µL helicase termination buffer consisting of 0.1 M Tris-HCl (pH 7.5), 20 mM EDTA, 0.5% SDS, 0.1% Nonidet P-40, 0.1% bromophenol blue, 0.1% xylene cyanol, and 25% glycerol. The terminated reaction mixture was subjected to native TBE 10% polyacrylamide gel electrophoresis. The radioactive RNAs in the gel were visualized with an Image Reader FLA-9000 (Fujifilm) and quantified by Multi Gauge V 3.11 software.

### 3.5. RNA Binding Assay

RNA binding to NS3 helicase was analyzed by gel mobility shift assay [40]. First, let-7 single-strand RNA (5'-UGAGGUAGUAGGUUGUAUAGU-3') was incubated with [γ-^32^P] ATP (Muromachi, Tokyo, Japan) and T4 polynucleotide kinase (Toyobo) at 37 °C for 60 min for labeling at the 5'-end of the single-strand RNA. The reaction mixture was subjected to phenol chloroform extraction for purification of labeled RNA. The reaction was carried out at room temperature for 15 min in 20 μL of the mixture consisting of 30 mM Tris-HCl (pH 7.5), 100 mM NaCl, 2 mM MgCl_2_, 1 mM DTT, 1 unit of RNasin Plus (Promega) per microliter, 300 nM NS3, 5 fmol let-7-labeled ssRNA, and an indicated concentration of SG1-23-1. The reaction was stopped by adding an equal volume of dye solution consisting of 0.025% bromophenol blue, 10% glycerol, and 0.5× Tris/borate/EDTA (TBE). The resulting mixture was subjected to native 6% polyacrylamide gel electrophoresis (acrylamide: bis acrylamide = 19:1). The radioactive RNA was visualized with the Image Reader FLA-9000 and quantified by Multi Gauge V 3.11 software.

### 3.6. Cell Lines

The following Huh-7-derived cell lines used in this study were maintained in Dulbecco’s modified Eagle’s medium containing 10% fetal calf serum and 0.5 mg/mL G418: The Lunet/Con1 LUN Sb #26 cell line, which harbors the subgenomic replicon RNA of the Con1 strain (genotype 1b) [34]; the Huh7/ORN3-5B #24 cell line, which harbors the subgenomic replicon RNA of the O strain (genotype 1b) [35]; the Huh7 Rep Feo cell line, which harbors the subgenomic replicon RNA of the N strain (genotype 1b) [33]; and the OR6 cell line, which harbors the full genomic RNA of the O strain (genotype 1b) [35].

### 3.7. Determination of Luciferase Activity in HCV Replicon Cells

HCV replicon cells were seeded at 2 × 10^4^ cells per well in a 48-well plate 24 h before treatment. The extract SG1-23-1 was added to the culture medium at various concentrations. The treated cells were harvested 72 h post-treatment and lysed in cell culture lysis reagent (Promega) or *Renilla* luciferase assay lysis buffer (Promega). Luciferase activity in the harvested cells was estimated with a luciferase assay system (Promega) or a *Renilla* luciferase assay system (Promega). The resulting luminescence was detected by the Luminescencer-JNR AB-2100 (ATTO, Tokyo, Japan) and corresponded to the expression level of the HCV replicon.

### 3.8. Determination of Cytotoxicity in HCV Replicon Cells

HCV replicon cells were seeded at a density of 1 × 10^4^ cells per well in a 96-well plate and incubated at 37 °C for 24 h. The extract fraction of the sample code SG1-23-1 was added to the culture medium at various concentrations. These cells were treated with an indicated concentration of the extract fraction and then were harvested 72 h post-treatment. Cell viability was measured by dimethylthiazol carboxymethoxy-phenylsulfophenyl tetrazolium (MTS) assay using a CellTiter 96 aqueous one-solution cell proliferation assay kit (Promega).

### 3.9. Effects on Activities of Luciferase and Internal Ribosome Entry Site (IRES)

The plasmid pEF Fluc IN and pEF Rluc EMCV IRES Feo were described previously [41]. The firefly luciferase gene was replaced with the *Renilla* luciferase gene in the plasmid pEF Fluc IN. The resulting plasmid was designated as pEF RlucIN in this study. The Huh7 cells were transfected with the pEF Fluc IN, pEF Rluc IN, or pEF Rluc EMCV IRES Feo and then were established in a medium containing 0.25 mg/mL G418 as described previously [41]. These cell lines were seeded at 2 × 10^4^ cells per well in a 48-well plate 24 h before treatment, treated with 50 μg/mL extract SG1-23-1, and then harvested at 72 h post-treatment. Activities of firefly and *Renilla* luciferases in pEF Rluc EMCV IRES Feo were measured with the dual luciferase reporter assay system (Promega). Total protein concentration was measured using the BCA Protein Assay Reagent Kit (Thermo Scientific, Rockford, IL, USA) to normalize luciferase activity.

### 3.10. Western Blotting

The cells were lysed in lysis buffer containing Cell Culture Lysis Reagent (Promega). The cell lysate was subjected to SDS-10% polyacrylamide gel (SDS-PAGE). The proteins in the gel were transferred onto a polyvinylidene fluoride (PVDF) membrane. The resulting membrane was incubated with the primary antibodies at 4 °C overnight and then was washed three times with PBS containing 0.02% Tween 20 (PBS-T). The resulting membrane was reacted with a horseradish peroxidase-labeled anti-IgG antibody at room temperature for 2 h and then was washed three times with PBS-T. The reacted proteins were visualized with ImmunoStar LD (Wako Pure Chemical, Osaka, Japan). The antibodies to NS3 (Abcam, Cambridge, UK), NS5A (ViroGen, Watertown, MA, USA) and beta-actin were purchased from New England Biolabs (Beverly, MA, USA) and were used as the primary antibodies in this study.

### 3.11. Reverse-Transcription Polymerase Chain Reaction (RT-PCR)

The previously described method of RT-PCR [41] was slightly modified, as described below. Total RNA was isolated from cultured cells with the RNAqueous-4PCR kit (Ambion, Austin, TX, USA) and then was reverse-transcribed with a Superscript III reverse transcriptase (Invitrogen, Carlsbad, CA, USA). The transcribed mRNA was amplified with PCR using AmpliTaq Gold DNA polymerase (Applied Biosystems, Foster City, CA, USA) and an appropriate primer pair. Primer sequences targeting the genes encoding 2',5'-oligoadenylate synthetase (2',5'-OAS), myxovirus resistance protein A (MxA), and glyceraldehyde-3-phosphate dehydrogenase (GAPDH) were described previously [41].

## 4. Conclusions

In conclusion, we showed that the ethyl acetate extract from *Alloeocomatella polycladia *significantly inhibits HCV replication by suppressing viral helicase activity. The purification of an inhibitory compound from the extract of *Alloeocomatella polycladia *will be required in order to improve the efficacy of chemical modification of the compound(s).
